# Structural and Functional Similarity between the Bacterial Type III Secretion System Needle Protein PrgI and the Eukaryotic Apoptosis Bcl-2 Proteins

**DOI:** 10.1371/journal.pone.0007442

**Published:** 2009-10-13

**Authors:** Matthew D. Shortridge, Robert Powers

**Affiliations:** Department of Chemistry, University of Nebraska-Lincoln, Lincoln, Nebraska, United States of America; Miami University, United States of America

## Abstract

**Background:**

Functional similarity is challenging to identify when global sequence and structure similarity is low. Active-sites or functionally relevant regions are evolutionarily more stable relative to the remainder of a protein structure and provide an alternative means to identify potential functional similarity between proteins. We recently developed the FAST-NMR methodology to discover biochemical functions or functional hypotheses of proteins of unknown function by experimentally identifying ligand binding sites. FAST-NMR utilizes our CPASS software and database to assign a function based on a similarity in the structure and sequence of ligand binding sites between proteins of known and unknown function.

**Methodology/Principal Findings:**

The PrgI protein from *Salmonella typhimurium* forms the needle complex in the type III secretion system (T3SS). A FAST-NMR screen identified a similarity between the ligand binding sites of PrgI and the Bcl-2 apoptosis protein Bcl-xL. These ligand binding sites correlate with known protein-protein binding interfaces required for oligomerization. Both proteins form membrane pores through this oligomerization to release effector proteins to stimulate cell death. Structural analysis indicates an overlap between the PrgI structure and the pore forming motif of Bcl-xL. A sequence alignment indicates conservation between the PrgI and Bcl-xL ligand binding sites and pore formation regions. This active-site similarity was then used to verify that chelerythrine, a known Bcl-xL inhibitor, also binds PrgI.

**Conclusions/Significance:**

A structural and functional relationship between the bacterial T3SS and eukaryotic apoptosis was identified using our FAST-NMR ligand affinity screen in combination with a bioinformatic analysis based on our CPASS program. A similarity between PrgI and Bcl-xL is not readily apparent using traditional global sequence and structure analysis, but was only identified because of conservation in ligand binding sites. These results demonstrate the unique opportunity that ligand-binding sites provide for the identification of functional relationships when global sequence and structural information is limited.

## Introduction

Functional regions of a protein are evolutionarily more stable relative to the remainder of the protein [1], [2]. The evolutionary correlation between ligand binding sites, ligand structure and protein function has also been demonstrated by a network of ligand-binding-site similarity described by Park & Kim [3]. A variety of computational methods have attempted to exploit the evolutionary stability of functional regions by identifying ligand binding sites as a method to predict function [4], [5]. Unfortunately, the combined requirements of predicting the ligand, the binding site, and a similarity to an annotated protein lead to a high level of ambiguity. Recently, we developed the Functional Annotation Screening Technology by Nuclear Magnetic Resonance (FAST-NMR) assay to experimentally identify ligand binding sites to annotate proteins of unknown function [5]–[7]. FAST-NMR utilizes the Comparison of Protein Active Site Structures (CPASS) software and database to identify similar sequence and structure characteristics between experimentally identified ligand binding sites for proteins of known and unknown function [8]. Applying the FAST-NMR method on previously annotated systems also enables experimental ligand binding site data to identify functional relationships that otherwise would not be recognized based solely on global sequence and structure similarity.

The type three secretion system (T3SS) is composed of 20–25 different proteins, which are assembled in a highly choreographed mechanism similar to the assembly of flagella [9]–[11]. In *Salmonella typhimurium*, the needle complex is responsible for puncturing a host's cell membrane to allow effector proteins (SipB, SipC, SipD) from *S. typhimurium* to enter the host [12]. Many of these effectors can activate bacterial induced apoptosis of a hosts' cell by interacting with capsase-1 [13] in a mechanism similar to apoptosis in eukaryotic cells [14]. The needle complex is a large homomultimer composed of ∼120 repeated copies of the monomeric protein PrgI, a small helical protein of 83 amino acids [15]. The monomeric form of PrgI is a helix-turn-helix motif with two symmetrically charged surfaces and a conserved loop region, PxxP domain, which are important for needle assembly [15]–[17]. The charged surfaces of PrgI responsible for needle assembly also provide a potential binding site for small molecule ligands. This makes PrgI an attractive drug target to disrupt the formation of the needle complex and prevent infection by *S. typhimurium*. However, to date there have been no reported ligands that bind to either region of this protein-protein interaction site.

The PrgI needle complex protein from *S. typhimurium* T3SS was screened in our FAST-NMR assay, which resulted in the identification of a functional similarity between the ligand binding sites of PrgI and the anti-apoptosis protein Bcl-xL. Additionally, Dali [18] and T-Coffee [19] analysis found regions of structure and sequence similarity between the two proteins consistent with the FAST-NMR results. The predicted active-site similarity between PrgI and Bcl-xL was also used to experimentally verify that chelerythrine [20], a ligand known to inhibit Bcl-xL and induce apoptosis, also binds PrgI. These results provide experimental evidence that suggest a functional relationship between the bacterial type III secretion systems and apoptosis. This is consistent with a general conservation in function between PrgI and the Bcl-2 family of proteins that includes Bcl-xL; both form membrane pores through oligomerization using a conserved helix-turn-helix motif to release effectors to stimulate cell death.

## Results

### Results from the FAST-NMR screen

The needle complex protein, PrgI, from *S. typhimurium* is an attractive antibacterial target because the protein is exposed to the cell surface and blocking this target could prevent injection of virulence factors into the host [21]. The interaction of PrgI with the host membrane stimulates the delivery of effectors from the bacteria into the host cytosol to induce cell death. Recently an NMR structure was determined for a monomeric form of PrgI [15], which enabled the screening of PrgI using the FAST-NMR assay [6]. FAST-NMR combines NMR ligand affinity screening [22] using a fragment-based functional library [23] with structural biology and bioinformatics [8] to rapidly determine protein-ligand complexes [24] and infer functional relationship between proteins based on similarities in functional epitopes. Also, the resulting protein-ligand co-structure provides a valuable starting point for structure-based drug design.

**Figure 1:**
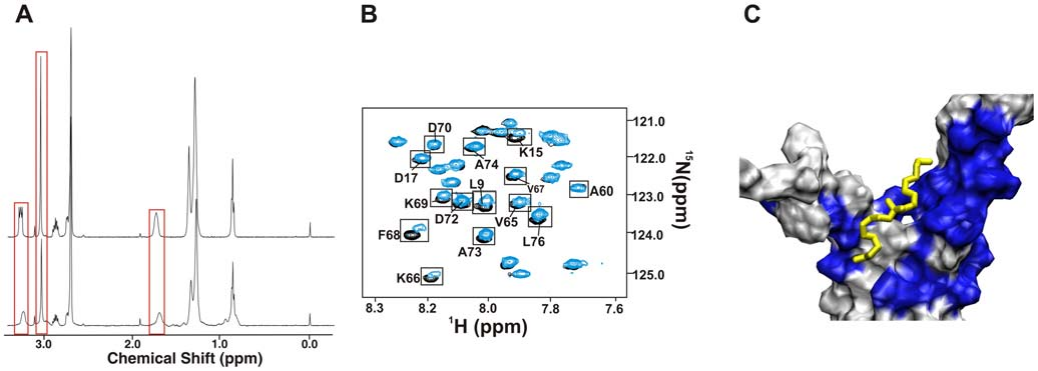
Identification of PrgI Binding Ligands. (A) DDAB NMR spectra in the absence (*top*) and presence (*bottom*) of PrgI illustrating changes in NMR intensities (boxed) upon binding PrgI. Both free and bound 1D ^1^H NMR spectra were normalized to a constant DMSO signal intensity. (B) Expanded view of the superimposed 2D ^15^N-^1^H HSQC spectra of the free and DDAB bound PrgI NMR samples. Residues that incur a chemical shift perturbation are boxed. (C) Expanded view of PrgI surface rendered in VMD [78] where residues that incur a chemical shift change are colored blue and DDAB is colored yellow. Co-structure based on NMR determined ligand binding site using AutoDock [27] and our AutoDockFilter program [24].

FAST-NMR applies a tiered approach to screening [22] to minimize resources and increase throughput (Figure 1). First, PrgI was screened with the functional chemical library using 1D ^1^H NMR line-broadening experiments. Five compounds (L-carnitine inner salt, didecyldimethylammonium bromide, 1-methylimidazole, methiothepin mesylate salt, sucrose) were found to bind PrgI by showing a significant decrease in ^1^H peak intensity upon addition of 25 µM of PrgI. This was determined by comparing normalized ^1^H ligand peak intensities between the free and bound NMR spectra (Figure 1A). However, the secondary 2D ^15^N-^1^H HSQC experiments identified the lipid derivative didecyldimethylammonium bromide (DDAB) as the only specific PrgI binder (Figure 1B) based on the observation of a significant number of chemical shift changes in the spectrum. The remaining four compounds elicited no change in chemical shifts in the PrgI 2D ^15^N-^1^H HSQC spectrum, which suggest the compounds bound non-specifically to PrgI. PrgI was found to bind DDAB with a *K_d_* of 553 µM as calculated by a 1D ^1^H line broadening method [25]. Finding a lipid derivative that specifically binds to PrgI is consistent with the protein's function; sensing new host cells and signaling secretion through an interaction with the host membrane [26].

Chemical shift perturbations (CSPs) in the 2D ^15^N-^1^H HSQC experiments between free PrgI and the complex identified the PrgI residues that bind DDAB. Mapping these CSPs onto the PrgI surface identified the DDAB binding site as corresponding to residues at the bifurcation point of the two helices (Figure 1C). Specifically, residues S6, L9, S13, K15, and D17 of helix 1 and N59, V65, K66, V67, F68, K69, D70, D72, A73 and L76 of helix 2 showed significant CSPs in the presence of DDAB as calculated by equation 1. This ligand binding site has been shown to be important for the formation of the T3SS needle complex in which PrgI forms a repeating coiled-coils structure [10]. According to recent alanine scanning and structural studies, the surface residues in the region between the bifurcation point of the two helices and the conserved loop region, PxxP domain, are important for needle assembly [15]–[17]. These residues bind to the backside of the bifurcation point of the two helices in a stacked N-terminus to C-terminus manner [15]–[17].

The PrgI residues exhibiting significant CSPs upon binding DDAB were used to guide and filter a molecular docking simulation based on our method to rapidly determine protein-ligand co-structures [24]. AutoDock 4.0 [27] was used to calculate 100 docked structures within a 3D grid defined by the CSPs. Our AutoDock Filter program (ADF) selected the best conformer based on consistency with the magnitude of chemical shift changes [24]. The ligand is expected to be closest to the protein residues that incurred the largest CSPs. The best PrgI-DDAB docked structure is shown in Figure 1C, where DDAB adopts an extended conformation that straddles both helices of PrgI.

### Analysis of CPASS and Structure Similarity Results

**Figure 2:**
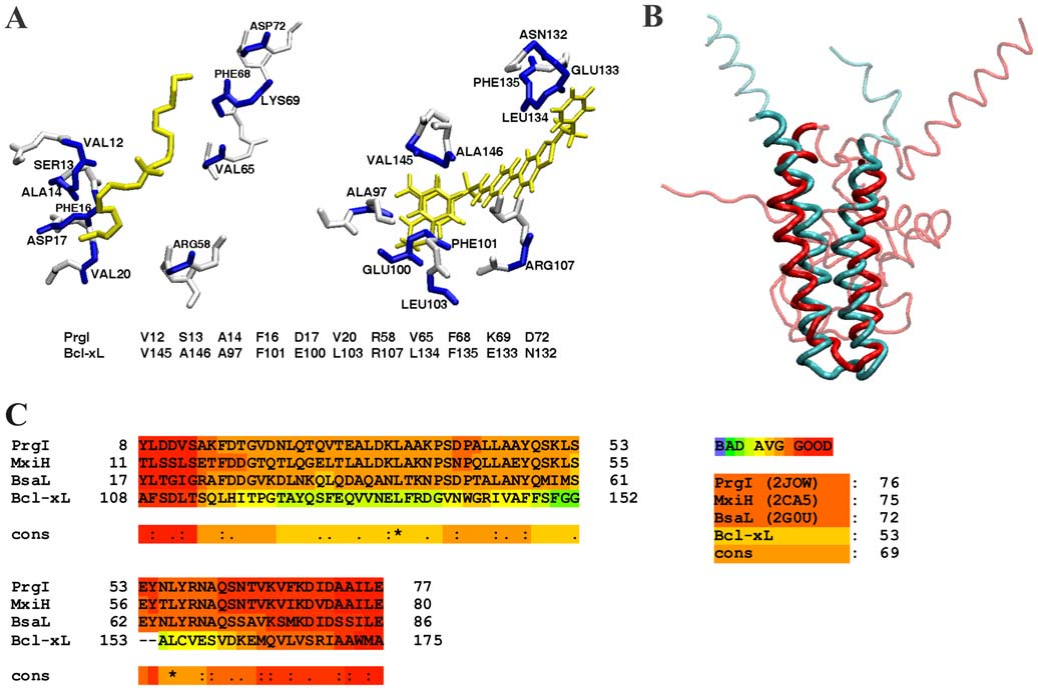
Active Site Similarity between PrgI and Bcl-xL. (A) CPASS alignment of the *S. typhimurium* PrgI active-site complexed to DDAB with the active-site of human Bcl-2 protein (Bcl-xL) complexed with acyl-sulfonamide-based inhibitor. The residues aligned by CPASS are labeled and colored blue in the structures. The active site sequence alignment is also shown below the structures. The ligands are colored yellow. (B) Overlay of the human Bcl-2 protein (red) with *S. typhimurium* PrgI (turquoise) based on a DaliLite [29] alignment. (C) Multiple-sequence alignment of the three known T3SS structures of *S. typhimurium* PrgI, *B. pseudomallei* BsaL, and *S. flexneri* MxiH with the human Bcl-2 protein (Bcl-xL). The reliability of the each amino acid alignment is color-coded from blue (poor) to red (good) using the CORE index [35]. The consensus alignment received a score of 69, where a perfect alignment receives a score of 100.

Comparison of Protein Active Site Structures (CPASS) analysis of the PrgI-DDAB complex identified a human Bcl-2 protein family member (the anti-apoptosis regulating protein Bcl-xL (PDB-ID:1YSN) complexed to an acyl-sulfonamide-based inhibitor (ABT-737)) [28] as the top hit based on a ligand binding-site CPASS similarity score of 37.7%. The CPASS alignment is shown in Figure 2A and is based on maximizing the spatial orientation of similar residue types between the two ligand-binding sites. All other proteins with a CPASS similarity >30% were also evaluated, but Bcl-xL was the only protein that gave a reliable CPASS score and showed some level of structure or sequence similarity to PrgI. It is important to note that the CPASS identified similarity between PrgI and Bcl-xL was fundamentally dependent on the existence of a Bcl-xL-ligand complex in the PDB. Ligand complexes for other members of the Bcl-2 protein family (Bax, Bid) currently do not exist.

While DDAB and ABT-737 are distinctly different ligands, the compounds share strong similarities in their mode of protein interactions. ABT-737 binds Bcl-xL edge-on in an elongated conformation where a minimal number of atoms contact the hydrophobic binding cleft of Bcl-xL. In this manner, DDAB mimics this edge contact interaction of ABT-737 with the similar hydrophobic binding cleft in PrgI. Also, ABT-737 binds in a protein-protein binding interface similar to DDAB, where inhibiting protein interactions is the drug's mechanism of action in cancer cells [28]. Thus, the PrgI and Bcl-xL ligand binding-sites are functionally similar.

A pairwise structure alignment using DaliLite [29] yielded a non-significant Z-score of 1.4 and only 6% sequence identity between PrgI (PDB ID:2JOW) and Bcl-xL (PDB ID:1YSN). Nevertheless, the helix-turn-helix structure of PrgI (residues S13-V65) overlaps the buried helix-turn-helix motif (N136-I182) in Bcl-xL that corresponds to helices α5 (residues W137-D156) and α6 (residues L162-D176) (Figure 2B). A focused pairwise comparison between the full PrgI protein and the α5 and α6 helices of Bcl-xL gave a low but significant Z-score of 3.3 with a root-mean-square-difference (rmsd) of 3.1 Å. The sequence identity also increases from 6% to 9% between the full and focused pairwise alignments, respectively.

While there is an overlap between the DaliLite alignment of PrgI with Bcl-xL and the protein ligand binding sites identified by CPASS, these sites are not identical. This arises because the CPASS similarity is not confined by the primary sequence of the two proteins, but simply captures the spatial orientation of conserved residues around a ligand binding site. This is illustrated by the non-sequential sequence alignment of the PrgI and Bcl-xL ligand binding sites in Figure 2. The exclusion of the sequence connectivity as a constraint to determine an alignment illustrates the advantage of CPASS in identifying a functional relationship and the limitation of global sequence and structure alignments such as Dali and BLAST [29], [30].

### Sequence Similarity Results

A BLAST [30] homology search using the PrgI and Bcl-xL sequences did not yield any significant information relating PrgI to Bcl-xL. The Bcl-xL sequence only identified homology to other Bcl-2 proteins. Similarly, the PrgI sequence was only aligned to other T3SS needle proteins. This is consistent with a ClustlW2 [31] sequence alignment between PrgI and Bcl-xL that resulted in a low 14.3% sequence similarity, which falls below the twilight zone of sequence similarity [32]. Also, focused BLAST searches did not provide any new information. Searching microbial genomes using the Bcl-2 sequences or searching the human genome with T3SS sequences did not identify any sequence alignments with significant E-values. Thus, global sequence alignments did not readily result in identifying any relationship between T3SS and apoptosis proteins. This highlights the power of active site similarity searches to identify potentially new functional similarities in proteins.

Hidden Markov model (HMM) methods [33] provide an alternative and more robust approach to identify homology between distantly related proteins with low sequence similarity relative to traditional BLAST searches. The T-Coffee web server (http://www.tcoffee.org/) provides a consensus sequence alignment (M-Coffee) using multiple HMM protocols [19]. A reliable alignment of conserved residues (Figure 2C) was obtained between the known T3SS structures of PrgI (PDB ID: 2JOW), BsaL (PDB ID: 2G0U) from *Burkholderia pseudomallei*, and MxiH (PDB ID: 2CA5) from *Shigella flexneri* with the human Bcl-xL (PDB ID: 1YSN) protein. The multiple-sequence alignment was obtained using EXPRESSO (3DCoffee) [34] that combines structural information with a HMM sequence alignment method. The reliability of the per residue alignment is color-coded using the CORE index (consistency of overall residue evaluation) [35], where the majority of residues were in the average to good range. The alignment of Bcl-xL with the three T3SS structures received a CORE index score of 53, where a score ≥50 indicates a 90% probability of being correctly aligned [19]. For comparison, the alignment of the three known human T3SS proteins resulted in a range of scores from 72 to 76. Thus, PrgI aligns preferentially to the other T3SS proteins, but its alignment to the pore forming helices in Bcl-xL is significant and reliable. Importantly, the sequence alignment of PrgI with Bcl-xL encompasses the same residues involved in the ligand binding sites identified by CPASS and the structural similarity identified by DaliLite.

### Identification of a Second PrgI Ligand Binding Site

The identification of a compound that binds similarly to both PrgI and Bcl-xL would further establish a functional relationship between these two proteins. The BindingDB [36] was used to identify potential inhibitors of PrgI based on the CPASS predicted active site similarity with Bcl-xL. A total of 71 ligands were reported to bind Bcl-xL. A majority of the compounds were piperazine derivatives and were not readily available. Two compounds, chelerythrine and sanquinarine were identified as having affinity to Bcl-xL and were both available from commercial suppliers. Chelerythrine was selected over sanquinarine based on previous NMR screening and docking studies that suggested chelerythrine binds between α4, α5 and α6 of Bcl-xL [37]. This region of Bcl-xL was predicted to overlap with PrgI based on the pairwise Dali alignment (Figure 2B). Conversely, sanquinarine bound the BH3 binding cleft of Bcl-xL and thus was not selected for this secondary binding analysis [37].

**Figure 3:**
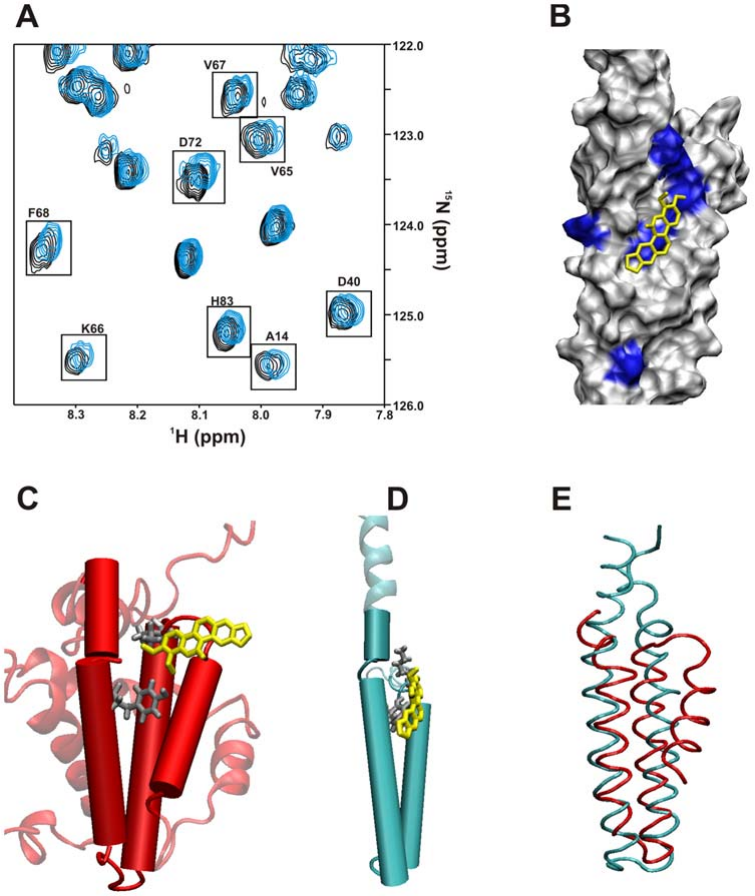
Verification that the Bcl-xL inhibitor chelerythrine also binds PrgI. (A). Expanded overlay of the 2D ^15^N-^1^H HSQC spectra for free PrgI (black) and PrgI bound to chelerythrine (blue). CSPs greater than one standard deviation are boxed. (B) An AutoDock/ADF docked structure of PrgI complexed with chelerythrine based on the observed CSPs from (A). (C) The Bcl-xL region shown to bind chelerythrine is highlighted while the remaining protein structure is transparent. Chelerythrine is colored yellow and is drawn with licorice bonds. Side-chains for Y173 and V135 are shown as licorice bonds and colored grey. (D) A ribbon diagram of the AutoDock/ADF docked PrgI-chelerythrine co-structure. The PrgI-chelerythrine binding region that overlaps with Bcl-xL is highlighted. Chelerythrine is colored yellow and is drawn with licorice bonds. Side-chains for Y57 and K15 are shown as licorice bonds and colored grey. (E) An expanded view of the overlay of Bcl-xL (red) with PrgI (blue) illustrating the structural similarity of the chelerythrine binding sites.

**Figure 4:**
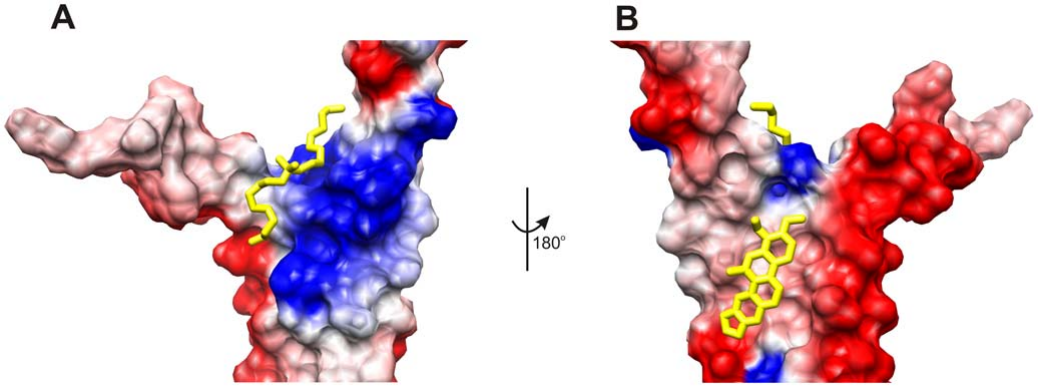
The two PrgI ligand binding sites identified using FAST-NMR. The two PrgI ligand binding sites are highlighted on an electrostatic potential surface (blue positive charge, red negative charge) calculated with DelPhi [79] implemented in Chimera [80]. The didecyldimethylammonium bromide binding site (A) is found in a region responsible for needle formation while the chelerythrine binding site (B) is found on the opposite face.

A comparison between the free and chelerythrine bound PrgI 2D ^15^N-^1^H HSQC spectra (Figure 3A) identified a chelerythrine binding site on PrgI (Figure 3B). The PrgI residues that exhibited chemical shift changes upon binding chelerythrine include residues A14, K15 in helix 1 and residues Y57, N59, A60, V65, K66, V67, F68, and D72 in helix 2. The AutoDock/ADF docked structure of PrgI with chelerythrine suggests PrgI residues K15 and Y57 are the most important residues for chelerythrine binding based on a close contact with the ligand (Figure 3B). Many of the residues that show significant CSPs for PrgI bound to chelerythrine overlap with the DDAB residues, however, the chelerythrine binding site is on the opposite face of PrgI (Figure 4). This indicates that there are two ligand binding sites on PrgI that is consistent with the two known protein-protein interaction sites for PrgI self-oligomerization. The chelerythrine AutoDock docking energy decreased significantly compared to DDAB, −0.43 to −5.29 kcal/mol, respectively.

The binding site of chelerythrine on PrgI is nearly identical to the binding site of chelerythrine to Bcl-xL (Figure 3C and 3D). In Bcl-xL, the chelerythrine binding site is described as being located in the BH groove of helix α4, α5 and α6, which is composed of residues F131, R132, V135, Y173 and H177 (Figure 3C) [37]. Pairwise structure analysis between PrgI and Bcl-xL shows that Y173 of Bcl-xL and Y57 on PrgI are overlapping residues and K15 from PrgI is proximal to V135 from Bcl-xL (Figure 3E). The primary difference between the two proteins is the lack of α-helix 4 in PrgI, where helix 4 of Bcl-xL appears to act as a ‘cap’ encasing the ligand and effecting its relative binding orientation. Chelerythrine binds flat in the PrgI binding site, while the compound points into the corresponding Bcl-xL binding site partially overlaying helix α4. Again, both of these structures are docked models based on NMR CSPs and require a high-resolution x-ray or NMR structure to confirm the conformation of the chelerythrine binding site. It is paramount to note that this similarity in chelerythrine binding between the two proteins would have not been discovered if it was not for the identification of the initial conserved ligand binding site between PrgI and Bcl-xL using the FAST-NMR method in combination with the CPASS database.

## Discussion

### Ligand Binding Similarity of the Bcl-2 Family of Proteins with PrgI

A structural and functional similarity between PrgI, a type three secretion system protein, and Bcl-xL, a member of the Bcl-2 family of proteins involved in eukaryotic apoptosis, was identified from a FAST-NMR ligand affinity screen in combination with a bioinformatic analysis. This association is fundamentally based on the similarity in ligand binding sites depicted in Figure 2A, where the conserved helix-turn-helix motif simply provides secondary support of a PrgI and Bcl-2 functional link. While similar active sites provide a measure of functional similarity, inferring homology based solely on the observation of a similar helix-turn-helix motif is questionable. The helix-turn-helix is a common motif and without a global sequence similarity, an evolutionary lineage based solely on active site similarity cannot be readily established. However, identifying similar ligand-binding sites between the two proteins does provide support that the proteins share a common function and are expected to bind similar ligands.

The initial identification of the conserved DDAB ligand binding site between Bcl-xL and PrgI was used to predict, test and confirm that chelerythrine binds PrgI in a similar manner to Bcl-xL. This further supports the structural and functional similarity between PrgI and Bcl-xL, but also demonstrates the utility of active site similarity as a predictive tool for ligand binding. Chelerythrine was only tested for PrgI binding because of the proposed active site similarity with Bcl-xL. Thus, these studies have identified the first known ligands to bind PrgI (DDAB and chelerythrine). Both ligand binding sites are associated with the functionally important PrgI self-oligomerization sites. Therefore, compounds based on either the DDAB or chelerythrine scaffold may disrupt PrgI oligomerization. These compounds may serve as valuable chemical leads to develop novel antibiotics. Additionally, since the ligands bind in separate locations on the PrgI surface (Figure 4), the compounds present two distinct approaches for developing drugs targeting PrgI. Unfortunately, because chelerythrine also binds Bcl-xL it is reasonable to expect that an antibiotic designed using chelerythrine as a scaffold may produce undesirable off-target side effects. This issue may be minimized or eliminated by simply improving the PrgI binding affinity for chelerythrine derivatives. This illustrates another important feature of our FAST-NMR protocol; active site similarity is a useful tool to predict potential side effects due to off target inhibition, in addition to predicting potential drug leads. While computational methods for predicting potential drug toxicity [38] are useful because of their speed, validation requires experimental methods such as the FAST-NMR approach.

### Functional Similarity of the Bcl-2 Family of Proteins with PrgI

The Bcl-2 family of proteins are essential for eukaryotic apoptosis; where Bcl-xL is responsible for repressing cell death activity [14]. The *in vivo* binding partners of Bcl-xL include the pro-apoptosis proteins Bax, Bak and Bid. It has been shown that expression levels of repressor (Bcl-xL) and pro-apoptosis proteins (Bax, Bak and Bid) are reciprocal in nature suggesting precise regulation of eukaryotic apoptosis [39]. A combination of mutational and structure based work has shown that the BH3 binding domain of Bcl-xL is critical for binding interactions with other Bcl-2 proteins and apoptosis regulation [39].

The structure of Bcl-xL very closely resembles the structures of Bax, Bid, Bcl-2, and other members of the Bcl-2 family of proteins, which all resemble pore-forming domains of bacterial toxins [40]–[42]. Bcl-2, Bcl-xL, Bax and the truncated active form of Bid (tBid) have all been shown to form pores in liposomes, but a similar cellular function has only been observed for Bax [39], [43], [44]. In healthy cells, Bax is a monomer in the cytosol. Many different apoptotic signals result in the transfer of Bax to the outer mitochondrial membrane where an interaction with Bid and the lipid membrane induces Bax to form a supramolecular opening in the outer mitochondrial membrane [45], [46]. This pore structure causes the release of pro-apoptotic factors from the mitochondria into the cytoplasm to induce cell death [47] and contains ∼22 copies of Bax with a diameter of ∼20 nm. The interaction of Bcl-xL with Bax prevents Bax induced cell death [48], where drugs that disrupt Bcl-xL interacting with Bcl-2 proteins are a promising form of cancer therapy [49]. Bcl-xL has been described as a dominant-negative version of Bax [50].

PrgI comprises the T3SS needle structure, which is formed by a PrgI homomultimer composed of ∼120 copies of the protein [9]–[11]. This needle structure senses and punctures host membranes forming a pore to transfer proteins to induce cell death in a mechanism similar to eukaryotic apoptosis [12]–[14]. A general conservation in function between PrgI and the Bcl-2 protein family is readily apparent; both form membrane pores via a helix-turn-helix motif through oligomerization to release effectors to stimulate cell death. Additionally, PrgI requires PrgJ for oligomerization into the needle [10] while Bax requires Bid to induce pore formation [46]. Thus, a protein interaction with other members of the Bcl-2 family is required to either promote (Bid) or inhibit (Bcl-xL) Bax oligomerization. It is also interesting that PrgI was found to bind to a lipid analog and lipids have been found to play a role in Bax oligomerization [46].

Importantly, the experimentally observed ligand-binding sites for both PrgI and Bcl-xL are functionally equivalent and within the conserved helix-turn-helix motif. Both sites correspond to functionally critical protein-protein interaction sites required for oligomerization and pore formation. The DDAB binding site on PrgI overlaps with key residues involved in PrgI oligomerization and needle assembly. Similarly, ABT-737 is an inhibitor of apoptosis and functions by inhibiting Bcl-xL protein interactions [51]. Thus, the similarity in the ligand-binding sites helps establish a functional link between the two proteins.

### Structural Similarity of the Bcl-2 Family of Proteins with PrgI

The Bax pore-forming domain is conserved in Bcl-xL, Bcl-2 and Bid [40], [52] and corresponds to the helix-turn-helix motif (helices α5 and α6) that was identified by CPASS to be similar to PrgI (Figure 2A). Also, a comparison of the Bcl-xL and PrgI structure by DaliLite resulted in the alignment of the PrgI structure with this conserved Bcl-2 helix-turn-helix motif (Figure 2B). Additionally, a multiple sequence alignment indicated a reliable similarity between T3SS needle-forming proteins and the Bcl-2 pore-forming region (Figure 2C). Thus, the PrgI structure can be viewed as a minimalistic version of the Bcl-2 structure, and it corresponds to the functionally essential and conserved core pore-forming domain.

Gene duplication along with insertion and/or deletions of sub-structures into variable genetic regions are known methods for the evolution of protein function [53], [54]. These processes may explain the evolution of the Bcl-2 family of proteins from a smaller PrgI-like ancestor. Since the PrgI structure overlaps with residues N136 to I182, this may suggest N- and C-terminal insertions generated a Bcl-2 protein from a PrgI-like ancestor. This is consistent with the hypothesis proposed by Aouacheria *et al.* [55], where the ancestral toxic pore forming domain (helices α5 and α6) required developing a means to prevent inappropriate apoptosis and to regulate cell death.

Presumably, a main function of the N- and C-terminal inserts into a PrgI-like ancestor would be to stabilize the monomer form of Bax until an apoptotic signal occurs. In effect, the insertions would provide a stronger control over the pore formation process. This is consistent with what has been experimentally observed, both the N- terminus and C-terminus residues of Bax are essential to maintain the monomer form of Bax in the cytosol [42], [56], [57]. Deletion of the first 20 amino acids from the N-terminus results in Bax being localized to the mitochondria [56], [57]. Similarly, the Bax structure indicates the C-terminal hydrophobic helix α9 is bent in a hydrophobic groove, but contains some critical solvent exposed polar residues that are necessary to maintain solubility [42]. In fact, a model for the translocation of Bax from the cytosol to the mitochondria requires a conformational change in Bax that opens up helix α9 and exposes the pore forming region composed of helices α5 and α6 [42], [58]. Deletions of 21 residues from the C-terminus, which includes part of helix α6, prevents oligomerization [59].

While Bax oligomerizes to form a circular pore structure containing ∼22 copies, this oligomerization process does not extend to form layers like the PrgI needle structure. The conformational change in Bax results in the globular domain remaining in the cytosol and sterically prevents oligomerization perpendicular to the membrane [60]. Thus, the structural insert that maintains a monomer Bax in the cytosol also prevents an unnecessary linear extension of the Bax oligomer out of the mitochondria membrane. Conversely, regulating PrgI oligomerization is not necessary since the assembly of the T3SS system is not detrimental to the cell. Therefore, a minimal pore-forming structure is all that is necessary for the T3SS system. The length of the PrgI needle is controlled by the proper assembly of the inner rod (PrgJ) that requires the InvJ protein [61]. The deletion of InvJ results in long non-functional needles.

### An Evolutionary Relationship between T3SS and Eukaryotic Apoptosis?

Based on the observed similarity in the structure and function between PrgI and the Bcl-2 protein family it is tempting to hypothesize that the proteins share a common ancestor. The structural comparison of PrgI with the Bcl-2 family of proteins discussed above suggests a possible evolutionary path. A common ancestral protein has been suggested for the Bcl-2 protein family, where pore formation using helices α5 and α6 is the ancestral protein's predicted primary function [55]. Similarly, T3SS are also predicted to evolve from a single gene [62] that is a simple but versatile export system [63]. Again, the helix-turn-helix is a common and ancient motif [64] demonstrating both its diverse utility and evolutionary stability. Thus, it is plausible that a simple and ancient PrgI-like protein could be an evolutionary precursor to both the Bcl-2 protein family and PrgI. It also appears unlikely that PrgI and the Bcl-2 protein family would evolve through a convergent process since the helix-turn-helix is such a simple and ancient motif [64] and essential to the function of both proteins. Evolving a readily available helix-turn-helix protein into either PrgI or the Bcl-2 protein family seems like a simpler path than the conversion of a uniquely distinct fold to incorporate a core helix-turn-helix motif. Also, the evolution of proteins from simple structural components has been previously proposed [65] and is consistent with other general evolutionary trends where complex systems evolve from simpler systems [66].

By analogy, the sharing of a common ancestor by PrgI and the Bcl-2 family of proteins would imply an evolutionary relationship between the T3SS and eukaryotic apoptosis systems. T3SS is a prime example of a vestigial system and an important illustration of the stepwise evolution of the flagella machinery [67], [68]. Therefore, it is reasonable to expect that other systems will be identified that share an evolutionary relationship with T3SS. T3SS is also an ancient system and clearly predates the origin of the mitochondria from prokaryote endosymbiosis [69], [70]. α-proteobacteria [69], which are close relatives of the mitochondria, are known to contain T3SS [63], [71], [72]. Could an obsolete T3SS system contribute valuable components to the eukaryotic apoptosis system after endosymbiosis? An evolutionary link has already been observed between a mitochondrial and T3SS protein [73], [74]. Furthermore, a detailed analysis of the origin of apoptotic proteins suggests a pivotal role for bacterial proteins in the evolution of eukaryotic apoptosis [75].

## Materials and Methods

### FAST-NMR Screen of PrgI

The *Salmonella typhimurium* type three secretion protein (T3SS) PrgI was screened with a functional library [23] using our FAST-NMR assay [5], [6]. Unlabeled and ^15^N labeled monomeric PrgI was graciously provided by Dr. Roberto DeGuzman (University of Kansas) along with the assigned ^15^N-^1^H HSQC spectrum. Sample preparation and experimental parameters for the NMR screening were executed in the same manner as described previously [6]. A total of 113 1D ^1^H NMR line-broadening spectra were collected to identify 5 binders from the functional chemical library of 437 compounds. Measurement of binding dissociation constants were completed as described previously [25]. Secondary 2D ^15^N-^1^H HSQC NMR experiments were collected only for the 5 compounds identified as binders in the line-broadening experiments. Chemical shift perturbations (CSPs) (eq 1) from the 2D ^15^N-^1^H HSQC NMR experiments were used to identify the PrgI ligand binding site, where only residues with a CSP greater than one standard deviation from the mean were used:
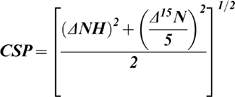
(1)where *ΔNH* is the difference between free and bound ^1^H amide chemical shifts (ppm) and *Δ15N* is the difference between free and bound ^15^N chemical shifts (ppm).

Our rapid approach to determine a ligand binding orientation was employed to determine a PrgI co-structure in the same manner as described previously [24]. The CSPs minimize the search space by using a significantly reduced AutoDock 3D grid. AutoDock 4.0 was used to generate 100 docked PrgI-ligand co-structures using the Lamarckian search algorithm with a population size of 300 and 500,000 energy evaluations [27]. Our AutoDockFilter (ADF) program then uses an NMR energy function based on the magnitude of CSPs to select the best ligand conformation. 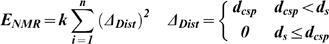
(2)where ADF calculates a pseudo-distance (*d_CSP_*) based on the magnitude of the NH CSP, which is then compared to the shortest distance (*d_S_*) between any atom in the residue that incurred an NH CSP and any atom in each docked ligand conformer. Comparison of these CSP-directed and selected ligand-docked structures with experimental x-ray and NMR structures has yielded an overall average rmsd of 1.17±0.74 Å [24].

A co-structure of the lipid derivative DDAB bound to PrgI was uploaded to the CPASS database (http://bionmr-c1.unl.edu/CPASS/cpass.htm) to identify proteins with similar ligand binding sites by maximizing an rmsd weighted BLOSUM62 [76], [77] scoring function (***S_ab_***). 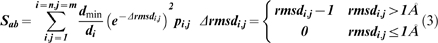
(3)where active site ***a*** contains ***n*** residues and is compared to active site ***b*** from the CPASS database which contains ***m*** residues, ***p_i,j_*** is the BLOSUM62 probability for amino-acid replacement for residue ***i*** from active site ***a*** with residue ***j*** from active site ***b***, ***Δ_i,j_*** is a corrected root-mean square difference in the Cα coordinate positions between residues ***i*** and ***j***, and ***d_min_/d_i_*** is the ratio of the shortest distance to the ligand among all amino-acids in the active site compared to the current amino-acid's shortest distance to the ligand. ***S_ab_*** is only summed over the optimal alignment for residue ***i*** from active site ***a*** with residue ***j*** from active site ***b***. It is not summed over all possible combinations of ***i*** and ***j***. If the number of residues are not identical between active sites ***a*** and ***b*** (n≠m), then the additional residues will not have a corresponding match. Each residue can only be used once in the alignment. If active site ***a*** contains unmatched residues, then no contribution is made to ***S_ab_*** which effectively reduces the maximal possible score that can be achieved for active site ***a***. Currently, there are ∼35,000 protein-ligand structures in the CPASS database. CPASS runs on a 16 node Beowulf Linux cluster, requires approximately 40 sec for each pair-wise comparison and takes ∼24 hrs to complete a full search against the entire database.

### Structure Similarity Searching

Native protein structures for PrgI (PDB ID: 2JOW) [15] and Bcl-xL (PDB ID: 1YSN) [28] were uploaded to the DaliLite [29] web server (http://www.ebi.ac.uk/DaliLite/) to identify regions of structure homology between the two proteins. To identify structure similarity and possible homologies between other proteins within the PDB, the structures were also uploaded to the full Dali [18] web server (http://www.ebi.ac.uk/dali/). A truncated version of the Bcl-xL structure was generated by identifying the amino acids within regions of structure similarity and removing these residues from the native PDB file. The truncated PDB file was searched for regions of similarity using the DaliLite web server (http://www.ebi.ac.uk/Tools/dalilite/index.html).

### Sequence Similarity Searching using BLAST and T-Coffee

Sequences from the T3SS and apoptosis regulation were downloaded from the NCBI server (http://www.ncbi.nlm.nih.gov/) and included PrgI (gi|16766179), InvJ (gi|16766198) and InvG (gi|474941) from *S. typhimurium*, and Bcl-xL, (gi|510901), Bak1 (gi|82571458), Bid (gi|4557361) and Bax (gi|231632) from *Homo sapiens* respectively. A full BLAST search was completed using these sequences associated with both systems as queries [30]. All BLAST sequence searches used default web settings. In addition, the sequences and structures for Bcl-xL (PDB-ID: 1YSN), *S. typhimurium* PrgI (PDB-ID: 2JOW), *B. pseudomallei* BsaL (PDB-ID: 2GOU) and *S. flexneri* MxiH (PDB-ID: 2CA5) obtained from the PDB were uploaded to the T-Coffee [19] web server (http://www.tcoffee.org/) to obtain a multiple sequence alignment using the EXPRESSO(3DCoffee) software [34]. Only the sequence region of the Bcl-xL structure that contained the pore-forming domain and yielded the highest alignment score was used for the multiple sequence alignment.

### Secondary Binding Site Similarity between Bcl-xL and PrgI

To further support a structural and functional similarity between Bcl-xL and PrgI, the BindingDB [36] (http://www.bindingdb.org/) was searched for commercially available compounds to test for binding to PrgI. The free ^15^N-^1^H HSQC spectrum was collected using 100 µM ^15^N labeled PrgI in 20 mM bis-Tris buffer with 100 mM sodium chloride at pH 7.0. A second PrgI sample was prepared in the same manner as above with the addition of 500 µM chelerythrine to generate the bound ^15^N-^1^H HSQC spectrum. Chemical shift perturbations and a PrgI-chelerythrine docked co-structure were determined as described previously [24] and was compared to the Bcl-xL-chelerythrine model [37].

## References

[1] Gerlt JA, Babbitt PC (2001). Divergent evolution of enzymatic function: mechanistically diverse superfamilies and functionally distinct suprafamilies.. Annu Rev Biochem.

[2] Mirny LA, Shakhnovich EI (1999). Universally conserved positions in protein folds: reading evolutionary signals about stability, folding kinetics and function.. J Mol Biol.

[3] Park K, Kim D (2008). Binding similarity network of ligand.. Proteins.

[4] Campbell SJ, Gold ND, Jackson RM, Westhead DR (2003). Ligand binding: functional site location, similarity and docking.. Curr Opin Struct Biol.

[5] Powers R, Mercier KA, Copeland JC (2008). The application of FAST-NMR for the identification of novel drug discovery targets.. Drug Discov Today.

[6] Mercier KA, Baran M, Ramanathan V, Revesz P, Xiao R (2006). FAST-NMR: functional annotation screening technology using NMR spectroscopy.. J Am Chem Soc.

[7] Powers R (2007). Functional genomics and NMR spectroscopy.. Comb Chem High Throughput Screen.

[8] Powers R, Copeland JC, Germer K, Mercier KA, Ramanathan V (2006). Comparison of protein active site structures for functional annotation of proteins and drug design.. Proteins.

[9] Cornelis GR, Van Gijsegem F (2000). Assembly and function of type III secretory systems.. Annu Rev Microbiol.

[10] Kimbrough TG, Miller SI (2002). Assembly of the type III secretion needle complex of Salmonella typhimurium.. Microbes Infect.

[11] Macnab RM (2003). How bacteria assemble flagella.. Annu Rev Microbiol.

[12] Galan JE (2001). Salmonella interactions with host cells: type III secretion at work.. Annu Rev Cell Dev Biol.

[13] Grassme H, Jendrossek V, Gulbins E (2001). Molecular mechanisms of bacteria induced apoptosis.. Apoptosis.

[14] Yan N, Shi Y (2005). Mechanisms of apoptosis through structural biology.. Annu Rev Cell Dev Biol.

[15] Wang Y, Ouellette AN, Egan CW, Rathinavelan T, Im W (2007). Differences in the electrostatic surfaces of the type III secretion needle proteins PrgI, BsaL, and MxiH.. J Mol Biol.

[16] Deane JE, Roversi P, Cordes FS, Johnson S, Kenjale R (2006). Molecular model of a type III secretion system needle: Implications for host-cell sensing.. Proc Natl Acad Sci U S A.

[17] Torruellas J, Jackson MW, Pennock JW, Plano GV (2005). The Yersinia pestis type III secretion needle plays a role in the regulation of Yop secretion.. Mol Microbiol.

[18] Dietmann S, Park J, Notredame C, Heger A, Lappe M (2001). A fully automatic evolutionary classification of protein folds: Dali Domain Dictionary version 3.. Nucleic Acids Res.

[19] Poirot O, O'Toole E, Notredame C (2003). Tcoffee@igs: A web server for computing, evaluating and combining multiple sequence alignments.. Nucleic Acids Res.

[20] Chan SL, Lee MC, Tan KO, Yang LK, Lee AS (2003). Identification of chelerythrine as an inhibitor of BclXL function.. J Biol Chem.

[21] Muller S, Feldman MF, Cornelis GR (2001). The Type III secretion system of Gram-negative bacteria: a potential therapeutic target?. Expert Opin Ther Targets.

[22] Mercier KA, Shortridge MD, Powers R (2009). A multi-step NMR screen for the identification and evaluation of chemical leads for drug discovery.. Comb Chem High Throughput Screen.

[23] Mercier KA, Germer K, Powers R (2006). Design and characterization of a functional library for NMR screening against novel protein targets.. Comb Chem High Throughput Screen.

[24] Stark J, Powers R (2008). Rapid protein-ligand costructures using chemical shift perturbations.. J Am Chem Soc.

[25] Shortridge MD, Hage DS, Harbison GS, Powers R (2008). Estimating protein-ligand binding affinity using high-throughput screening by NMR.. J Comb Chem.

[26] Kubori T, Sukhan A, Aizawa SI, Galan JE (2000). Molecular characterization and assembly of the needle complex of the Salmonella typhimurium type III protein secretion system.. Proc Natl Acad Sci U S A.

[27] Morris GM, Goodsell DS, Halliday RS, Huey R, Hart WE (1998). Automated docking using a Lamarckian genetic algorithm and an empirical binding free energy function.. J Comput Chem.

[28] Oltersdorf T, Elmore SW, Shoemaker AR, Armstrong RC, Augeri DJ (2005). An inhibitor of Bcl-2 family proteins induces regression of solid tumours.. Nature.

[29] Holm L, Park J (2000). DaliLite workbench for protein structure comparison.. Bioinformatics.

[30] Altschul SF, Madden TL, Schaffer AA, Zhang J, Zhang Z (1997). Gapped BLAST and PSI-BLAST: a new generation of protein database search programs.. Nucleic Acids Res.

[31] Larkin MA, Blackshields G, Brown NP, Chenna R, McGettigan PA (2007). Clustal W and Clustal X version 2.0.. Bioinformatics.

[32] Rost B (1999). Twilight zone of protein sequence alignments.. Protein Eng.

[33] Bystroff C, Krogh A (2008). Hidden Markov Models for prediction of protein features.. Methods Mol Biol.

[34] Armougom F, Moretti S, Poirot O, Audic S, Dumas P (2006). Expresso: automatic incorporation of structural information in multiple sequence alignments using 3D-Coffee..

[35] Notredame C, Abergel C, Andrade MA, (2003). Using multiple alignment methods to assess the quality of genomic data analysis.. Bioinformatics and genomes: current perspectives.

[36] Liu T, Lin Y, Wen X, Jorissen RN, Gilson MK (2007). BindingDB: a web-accessible database of experimentally determined protein-ligand binding affinities.. Nucleic Acids Res.

[37] Zhang YH, Bhunia A, Wan KF, Lee MC, Chan SL (2006). Chelerythrine and sanguinarine dock at distinct sites on BclXL that are not the classic BH3 binding cleft.. J Mol Biol.

[38] Xie L, Li J, Bourne PE (2009). Drug discovery using chemical systems biology: identification of the protein-ligand binding network to explain the side effects of CETP inhibitors.. PLoS Comput Biol.

[39] Chao DT, Korsmeyer SJ (1998). BCL-2 family: regulators of cell death.. Annu Rev Immunol.

[40] Chou JJ, Li H, Salvesen GS, Yuan J, Wagner G (1999). Solution structure of BID, an intracellular amplifier of apoptotic signaling.. Cell.

[41] McDonnell JM, Fushman D, Milliman CL, Korsmeyer SJ, Cowburn D (1999). Solution structure of the proapoptotic molecule BID: a structural basis for apoptotic agonists and antagonists.. Cell.

[42] Suzuki M, Youle RJ, Tjandra N (2000). Structure of Bax: coregulation of dimer formation and intracellular localization.. Cell.

[43] Vander Heiden MG, Thompson CB (1999). Bcl-2 proteins: regulators of apoptosis or of mitochondrial homeostasis?. Nat Cell Biol.

[44] Yan L, Miao Q, Sun Y, Yang F (2003). tBid forms a pore in the liposome membrane.. FEBS Lett.

[45] Annis MG, Soucie EL, Dlugosz PJ, Cruz-Aguado JA, Penn LZ (2005). Bax forms multispanning monomers that oligomerize to permeabilize membranes during apoptosis.. Embo J.

[46] Kuwana T, Mackey MR, Perkins G, Ellisman MH, Latterich M (2002). Bid, Bax, and lipids cooperate to form supramolecular openings in the outer mitochondrial membrane.. Cell.

[47] Breckenridge DG, Xue D (2004). Regulation of mitochondrial membrane permeabilization by BCL-2 family proteins and caspases.. Curr Opin Cell Biol.

[48] Fletcher JI, Meusburger S, Hawkins CJ, Riglar DT, Lee EF (2008). Apoptosis is triggered when prosurvival Bcl-2 proteins cannot restrain Bax.. Proc Natl Acad Sci U S A.

[49] Kang MH, Reynolds CP (2009). Bcl-2 Inhibitors: Targeting Mitochondrial Apoptotic Pathways in Cancer Therapy.. Clin Cancer Res.

[50] Billen LP, Kokoski CL, Lovell JF, Leber B, Andrews DW (2008). Bcl-XL inhibits membrane permeabilization by competing with Bax.. PLoS Biol.

[51] Wendt MD (2008). Discovery of ABT-263, a Bcl-family protein inhibitor: observations on targeting a large protein-protein interaction.. Expert Opin Drug Discovery.

[52] Muchmore SW, Sattler M, Liang H, Meadows RP, Harlan JE (1996). X-ray and NMR structure of human Bcl-xL, an inhibitor of programmed cell death.. Nature.

[53] Jiang H, Blouin C (2007). Insertions and the emergence of novel protein structure: a structure-based phylogenetic study of insertions.. BMC Bioinformatics.

[54] Morett E, Bork P (1998). Evolution of new protein function: recombinational enhancer Fis originated by horizontal gene transfer from the transcriptional regulator NtrC.. FEBS Lett.

[55] Aouacheria A, Brunet F, Gouy M (2005). Phylogenomics of life-or-death switches in multicellular animals: Bcl-2, BH3-Only, and BNip families of apoptotic regulators.. Mol Biol Evol.

[56] Cartron PF, Oliver L, Martin S, Moreau C, LeCabellec MT (2002). The expression of a new variant of the pro-apoptotic molecule Bax, Baxpsi, is correlated with an increased survival of glioblastoma multiforme patients.. Hum Mol Genet.

[57] Goping IS, Gross A, Lavoie JN, Nguyen M, Jemmerson R (1998). Regulated targeting of BAX to mitochondria.. J Cell Biol.

[58] Desagher S, Osen-Sand A, Nichols A, Eskes R, Montessuit S (1999). Bid-induced conformational change of Bax is responsible for mitochondrial cytochrome c release during apoptosis.. J Cell Biol.

[59] Er E, Lalier L, Cartron PF, Oliver L, Vallette FM (2007). Control of Bax homodimerization by its carboxyl terminus.. J Biol Chem.

[60] Lalier L, Cartron PF, Juin P, Nedelkina S, Manon S (2007). Bax activation and mitochondrial insertion during apoptosis.. Apoptosis.

[61] Marlovits TC, Kubori T, Lara-Tejero M, Thomas D, Unger VM (2006). Assembly of the inner rod determines needle length in the type III secretion injectisome.. Nature.

[62] Liu R, Ochman H (2007). Stepwise formation of the bacterial flagellar system.. Proc Natl Acad Sci U S A.

[63] Gophna U, Ron EZ, Graur D (2003). Bacterial type III secretion systems are ancient and evolved by multiple horizontal-transfer events.. Gene.

[64] Rosinski JA, Atchley WR (1999). Molecular evolution of helix-turn-helix proteins.. J Mol Evol.

[65] Kannan N, Neuwald AF (2005). Did Protein Kinase Regulatory Mechanisms Evolve Through Elaboration of a Simple Structural Component?. J Mol Biol.

[66] Shapiro R (2006). Small molecule interactions were central to the origin of life.. Q Rev Biol.

[67] McCann HC, Guttman DS (2008). Evolution of the type III secretion system and its effectors in plant-microbe interactions.. New Phytol.

[68] Gophna U, Ron EZ, Graur D (2003). Bacterial type III secretion systems are ancient and evolved by multiple horizontal-transfer events.. Gene.

[69] Gray MW, Burger G, Lang BF (2001). The origin and early evolution of mitochondria.. GenomeBiology [online computer file].

[70] Koonin EV, Aravind L (2002). Origin and evolution of eukaryotic apoptosis: the bacterial connection.. Cell Growth Differ.

[71] Gauthier A, Thomas NA, Finlay BB (2003). Bacterial Injection Machines.. J Biol Chem.

[72] Batut J, Andersson SGE, O'Callaghan D (2004). The evolution of chronic infection strategies in the a-proteobacteria.. Nat Rev Microbiol.

[73] Pallen Mark J, Bailey Christopher M, Beatson Scott A (2006). Evolutionary links between FliH/YscL-like proteins from bacterial type III secretion systems and second-stalk components of the FoF1 and vacuolar ATPases.. Protein Sci.

[74] Mulkidjanian AY, Makarova KS, Galperin MY, Koonin EV (2007). Inventing the dynamo machine: the evolution of the F-type and V-type ATPases.. Nat Rev Microbiol.

[75] Koonin EV, Aravind L (2002). Origin and evolution of eukaryotic apoptosis: the bacterial connection.. Cell Death Differ.

[76] Henikoff S, Henikoff JG (1992). Amino acid substitution matrices from protein blocks.. Proc Natl Acad Sci U S A.

[77] Henikoff S, Henikoff JG (1993). Performance evaluation of amino acid substitution matrices.. Proteins.

[78] Humphrey W, Dalke A, Schulten K (1996). VMD: visual molecular dynamics.. J Mol Graph.

[79] Nicholls A, Honig B (1991). A rapid finite difference algorithm, utilizing successive over-relaxation to solve the Poisson-Boltzmann equation.. J Comput Chem.

[80] Pettersen EF, Goddard TD, Huang CC, Couch GS, Greenblatt DM (2004). UCSF Chimera–a visualization system for exploratory research and analysis.. J Comput Chem.

